# The Potential Protective Effect of *Physalis peruviana* L. against Carbon Tetrachloride-Induced Hepatotoxicity in Rats Is Mediated by Suppression of Oxidative Stress and Downregulation of MMP-9 Expression

**DOI:** 10.1155/2014/381413

**Published:** 2014-04-27

**Authors:** Ebtisam M. Al-Olayan, Manal F. El-Khadragy, Ahmed M. Aref, Mohamed S. Othman, Rami B. Kassab, Ahmed E. Abdel Moneim

**Affiliations:** ^1^Zoology Department, Faculty of Science, King Saud University, Riyadh 11451, Saudi Arabia; ^2^Zoology and Entomology Department, Faculty of Science, Helwan University, Cairo 11795, Egypt; ^3^Biological Science Department, Faculty of Dentistry, Modern Sciences and Arts (MSA) University, Giza 12111, Egypt; ^4^Biochemistry and Molecular Biology Department, Faculty of Biotechnology, Modern Sciences and Arts (MSA) University, Giza 12111, Egypt; ^5^Experimental Biology Department, Faculty of Science, Masaryk University, 62500 Brno, Czech Republic; ^6^Biochemistry and Molecular Biology Department, Asturias Institute of Biotechnology, University of Oviedo, 33006 Oviedo, Spain

## Abstract

The active constituent profile in Cape gooseberry (*Physalis peruviana* L.) juice was determined by GC-MS. Quercetin and kaempferol were active components in the juice. In this study we have evaluated its potential protective effect on hepatic injury and fibrosis induced by carbon tetrachloride (CCl_4_). Twenty-eight rats divided into 4 groups: Group I served as control group, and Group II received weekly i.p. injection of 2 mL CCl_4_/kg bwt for 12 weeks. Group III were supplemented with Physalis juice via the drinking water. The animals of Group IV received Physalis juice as Group III and also were intraperitoneally injected weekly with 2 mL CCl_4_/kg bwt for 12 weeks. Hepatoprotective effect was evaluated by improvement in liver enzymes serum levels, reduction in collagen areas, downregulation in expression of the fibrotic marker MMP-9, reduction in the peroxidative marker malonaldehyde and the inflammatory marker nitric oxide, and restoration of the activity of antioxidant enzymatic and nonenzymatic systems, namely, glutathione content, superoxide dismutase, catalase, glutathione-S-transferase, glutathione peroxidase, and glutathione reductase activities. The results show that the potential hepatoprotective effects of *Physalis peruviana* may be due to physalis acts by promotion of processes that restore hepatolobular architecture and through the inhibition of oxidative stress pathway.

## 1. Introduction

Liver diseases are amongst the most serious health problems in the world today and their prevention and treatment options still remain limited despite tremendous advances in modern medicine. The pathogenesis of hepatic diseases as well as the role of oxidative stress and inflammation therein is well established and, accordingly, blocking or retarding the chain reactions of oxidation and inflammation process could be promising therapeutic strategies for prevention and treatment of liver injury [1].

Vitaglione et al. [2] suggested that reactive oxygen species (ROS) including superoxide and hydroxyl radicals are known to play an important role in liver disease's pathology and progression and have been proven to associate with the intoxication by CCl_4_. Documented evidences suggested that CCl_4_ has been commonly used as hepatotoxins in experimental hepatopathy [3]. The first metabolite of CCl_4_ trichloromethyl free radical is believed to initiate the biochemical processes leading to oxidative stress, which is the direct cause of many pathological conditions such as diabetes mellitus, cancer, hypertension, kidney and liver damages, and even death [4, 5].

In recent years, considerably clinical and experimental evidences show that oxidative stress caused by an imbalance between the oxidant and antioxidant systems of the body in favor of the oxidants should be a major apoptotic stimulus in the different types of acute and chronic liver injury and hepatic fibrosis [6].

Hepatic fibrosis induced by CCl_4_ is associated with the exacerbation of lipid peroxidation and the depletion of antioxidant status [7]. Accordingly, successful antioxidant interventions, which to date has attracted intensive interests from investigators, offer insights into delaying or preventing occurrence and development of hepatic fibrosis and may be a potential and effective therapeutic strategy for prevention and treatment of hepatic fibrosis [8].

There are a number of evidences indicating that natural substances from edible and medicinal plants exhibited strong antioxidant activity that could act against hepatic toxicity caused by various toxicants [9, 10]. One of those candidate plants is Cape gooseberry (*Physalis peruviana* L.). Various bioactive compounds (withanolides and phenolics) are reported to be present in physalis [11]. Some of these compounds have a strong antioxidant property and prevent peroxidative damage to liver microsomes and hepatocytes [12].

We aimed in this study to evaluate the potential hepatoprotective effect of* Physalis peruviana* against CCl_4_-induced hepatotoxicity and liver fibrosis in rats.

## 2. Materials and Methods

### 2.1. Chemicals

Carbon tetrachloride (CCl_4_) and Tris-HCl buffer were purchased from Sigma (St. Louis, MO, USA). Perchloric acid, thiobarbituric acid (TBA), and trichloroacetic acid (TCA) were purchased from Merck. All other chemicals and reagents used in this study were of analytical grade. Double-distilled water was used as the solvent.

### 2.2. Animals

Adult male Wistar albino rats weighing 200–250 g (8–10 weeks) were obtained from the Holding Company for Biological Products and Vaccines (VACSERA, Cairo, Egypt). The animals were kept in wire bottomed cages in a room under standard condition of illumination with a 12-hour light-dark cycle 55 + 5% relative humidity and at 25 ± 2°C for one week until the beginning of treatment. They were provided with tap water and balanced diet* ad libitum*. All animals have received human care in compliance with the state authorities following the Egyptian rules of animal protection.

### 2.3. Plant Material

*Physalis peruviana* L. fresh fruits were collected from market of East Cairo, Egypt, in the months of February-March, 2012. The plant material was authenticated in Botany Department, Faculty of Science, Helwan University, Cairo, Egypt, on the basis of taxonomic characters and by direct comparison with the herbarium specimens available at the herbarium of the Botany Department.

### 2.4. Physalis Juice Preparation and Stability

The fresh fruits of* Physalis peruviana* L. (10 kg) were separated from their calyxes and homogenized. The pulp was filtered off, the filtrate was clear and yellow in colour, and then the filtrate was immediately diluted with distilled water in ratio 1 : 5 (V/V) and stored at 4°C for no longer than 2 months. Physalis juice stability was assessed by measuring initial total phenolic and flavonoid contents and evaluating the alterations after 2 and 3 days of exposure to the same conditions as the juice supplied to the animals.

### 2.5. Measurement of Flavonoids, Total Polyphenols, and* In Vitro* Free Radical Scavenging Assays

#### 2.5.1. Determination of Total Phenols

The total polyphenolic contents (TPC) were measured using Folin-Ciocalteau reagent based on the oxidation of polyphenols to a blue colored complex with an absorbance maximum of 750 nm. Calibration curve was prepared using gallic acid as standard for TPC which was measured as mg gallic acid equivalents (GAE) per milliliter of the sample (*μ*g/mL).

#### 2.5.2. Determination of Flavonoid Content

For the assessment of flavonoids a colorimetric method was used. Briefly, 1.50 mL of the deionized water was added to 0.25 mL of the sample and then 90 uL of 5% sodium nitrite (NaNO_2_) was added. Six min later, after addition of 180 uL of 10% AlCl_3_, mixture was allowed to stand for another 5 min before adding 0.6 mL of 1 M NaOH. By adding deionized water and mixing well, final volume was made up to 3 mL. The absorbance was measured at a fixed wavelength 510 nm. Calibration curve was prepared using quercetin as standard for total flavonoids which was measured as mg quercetin equivalents (QE) per milliliter of the sample (*μ*g/mL).

#### 2.5.3. Determination of DPPH Radical Scavenging Activity

The free radical scavenging capacity was evaluated by the 2, 2-diphenyl -1-picrylhydrazyl (DPPH) assay. In its radical form, DPPH is monitored at 517 nm but, upon reduction by an antioxidant or a radical species, the absorption decreases. Briefly, 1 mL of 0.25 mM solution of DPPH in methanol was added to 50, 100, 150, and 200 uL of sample in 950, 900, 850, and 800 *μ*L methanol, respectively. After 20 min, the absorbance was measured. Ascorbic acid was used as a positive control. The percentage DPPH decolorisation of the sample was calculated by the equation, % DPPH scavenging = [(*A*_control_ − *A*_sample_)/*A*_control_] − 100, where *A* is the absorbance.

#### 2.5.4. Determination of Superoxide Anion Scavenging Activity

The superoxide anion scavenging activity was determined by the method of Nishikimi et al. [13]. Superoxide anion derived from dissolved oxygen by a phenazine methosulfate (PMS)/NADH coupling reaction reduces nitroblue tetrazolium (NBT), which forms a violet colored complex. A decrease in color after addition of the antioxidant is a measure of its superoxide scavenging activity. To the reaction mixture containing phosphate buffer (100 mM, pH 7.4), NBT (1 mM) solution, NADH (1 mM), 50, 100, 150, and 200 uL of sample in 950, 900, 850, and 800 *μ*L methanol, respectively, and 1 mL of 1 mM PMS were added. After incubation at 25°C for 5 min, the absorbance was measured at 560 nm against a blank. Vit. C was used as a positive control.

#### 2.5.5. Determination of Nitric Oxide Radical Inhibition Activity

The nitric oxide radical inhibition activity was measured using Griess reagent. Briefly, sodium nitroprusside (10 mM) in phosphate buffered saline was mixed with 50, 100, 150, and 200 uL of sample in 950, 900, 850, and 800 *μ*L methanol, respectively, and incubated at room temperature for 150 min followed by addition of 0.5 mL of Griess reagent (1% sulfanilamide, 2% H_3_PO_4,_ and 0.1% N-(1-naphthyl)ethylenediamine dihydrochloride). The absorbance of the chromophore formed was read at 546 nm.

#### 2.5.6. Determination of Total Antioxidant Potential Activity

The total antioxidant potential was measured by the ability of sample to scavenge thiobarbituric acid reactive substances (TBARS) [14]. Briefly, 50, 100, 150, and 200 uL of the different samples were added to the 10% liver homogenate. Lipid peroxidation was initiated by addition of 100 *μ*L of 15 mM FeSO_4_ solution to 3 mL of liver homogenate (final concentration was 0.5 mM). After 30 min, 100 *μ*L of this reaction mixture was taken in a tube containing 1.5 mL of 0.67% thiobarbituric acid (TBA) in 50% acetic acid. Samples were incubated at 37°C for 1 hr, and then lipid peroxidation was measured using the reaction with TBA. The absorbance of the organic layer was measured at 532 nm. All reactions were carried out in triplicate. Vitamin C (Vit. C) was used as the positive control. The percentage of inhibition of lipid peroxidation was calculated, by the formula Inhibition (%) = (*A*_control_ − *A*_sample_) × 100/*A*_control_.

### 2.6. Gas Chromatography-Mass Spectrometry (GC-MS) Analysis

The GC-MS analysis of physalis juice was performed with Thermo Scientific, Trace GC Ultra & ISQ Single Quadruple MS. The inert gas helium (99.9995%) was used as carrier gas, at flow rate of 1.5 mL/min; split ratio 10 : 1; sample size, 1 *μ*L injected using the splitless injection technique; fused capillary silica column TG-5MS (30 m × 0.25 mm × 0.25 *μ*m). Temperatures: injector: 260°C, detector: 300°C, column: 70°C, 10°C min^−1^, and 260°C (10 min). The total GC running time is at 60 min. The MS was taken at 70 eV. The MS scan parameters included a mass range of m/z 40–1000, a scan interval of 0.5 s, a scan speed of 2000 amu s^−1^, and a detector voltage of 1.0 kV. Identification of compounds was conducted using the database of Wiley9 combined with NIST 11 mass spectral database. The name, molecular weight, molecular formula, and area under peak of the components of the test materials were ascertained.

### 2.7. Experimental Protocol

To study the protective effects of physalis juice on carbon tetrachloride mediated liver toxicity, twenty eight adult male rats were randomly allocated to four groups, seven rats of each. Group I (Con) served as control and received 300 *μ*L of saline by intraperitoneal (i.p.) injection route each week. Group II (CCl_4_) received weekly i.p. injection of 2 mL CCl_4_/kg bwt for 12 weeks as described by Sohn et al. [15]. Group III (physalis) received juice supplied on dark water bottles and renewed every 2-3 days. The animals of Group IV (physalis + CCl_4_) received physalis juice as Group III and were also intraperitoneally injected weekly with 2 mL CCl_4_/kg bwt for 12 weeks. After one week of the last i.p. injection of CCl_4_, the animals of all groups were anesthetized with chloroform and blood sampling was performed by cardiac puncture. The collected blood samples were allowed to clot for half an hour at 8°C. Serum was separated by centrifugation at 3000 rpm for 15 min at 4°C to separate serum, stored at −20°C, and used for the estimation of marker enzymes, namely, alanine aminotransferase (ALT), aspartate aminotransferase (AST), *γ*-glutamyl transpeptidase (*γ*GT), alkaline phosphatase (ALP), and total bilirubin (TB). The livers were dissected out immediately, washed, and homogenized immediately to give 50% (w/v) homogenate in ice-cold medium containing 50 mM Tris-HCl, pH, 7.4. The homogenate was centrifuged at 3000 rpm for 10 min at 4°C. The supernatant (10%) was used for the various biochemical determinations.

### 2.8. Histopathological Examination

Tissue samples were fixed in 10% neutral formalin for 24 h and paraffin blocks were obtained and routinely processed for light microscopy. Slices of 4-5 *μ*m were obtained from the prepared blocks and stained with hematoxylin and eosin as well as Sirius Red for hepatic fibrosis. The preparations obtained were visualized using a Nikon microscopy at a magnification of 400×.

### 2.9. Liver Function Tests

Colorimetric determination of alanine aminotransferase or aspartate aminotransferase, ALT or AST, was estimated by measuring the amount of pyruvate or oxaloacetate produced by forming 2, 4-dinitrophenylhydrazine according to the method of Reitman and Frankel [16]. Glutamyl transferase and alkaline phosphatase, *γ*GT and ALP, were assayed using kits provided by Randox Laboratories Co. according to the methods described by Szasz [17] and Belfield and Goldberg [18], respectively. Also, total bilirubin, TB, in serum was assayed according to the method of Garber [19].

### 2.10. Oxidative Stress Markers

Serum and homogenates of liver were used to determine malondialdehyde (MDA) as indicator of lipid peroxidation by reaction of thiobarbituric acid according to the method of Ohkawa et al. [20], nitrite/nitrate (nitric oxide, NO) was measured using the method of Green et al. [21] and glutathione (GSH) was measured as described by Ellman [22].

### 2.11. Enzymatic Antioxidant Status

Homogenates of liver were used for determination of superoxide dismutase (SOD) as described by Nishikimi et al. [13], catalase (CAT) as described by Aebi [23], glutathione peroxidase (GPx) as described by Paglia and Valentine [24], glutathione-S-transferase (GST) as described by Habig et al. [25], and glutathione reductase (GRd) as described by Factor et al. [26].

### 2.12. Real Time PCR

Total RNA was isolated from the liver tissue using an RNeasy Plus Minikit (Qiagen, Valencia, CA). One microgram total RNA and random primers were used for cDNA synthesis using the RevertAid H Minus Reverse Transcriptase (Fermentas, Thermo Fisher Scientific Inc., Canada). For real time PCR analysis, the cDNA samples are run in triplicate and *β*-actin is used as reference gene. Each PCR amplification includes nontemplate controls containing all reagents except cDNA. Real time PCR reactions were performed using Power SYBR Green (Life Technologies, CA) and was conducted on the Applied Biosystems 7500 Instrument. The typical thermal profile is 95°C for 3 min, followed by 40 cycles of 95°C for 15 s and 56°C for 30 s. After PCR amplification, the ΔCt is calculated by subtraction of the *β*-actin Ct from each sample Ct. The method of Pfaffl was used for data analysis. The PCR primers for INOS, GPx, and GRd genes were synthesized by Jena Bioscience GmbH (Jena, Germany). Primers were designed using Primer-Blast program from NCBI. For a reference gene, the *β*-actin is used. The primer sets used the following.

 iNOS (S): 5′-GAAAGAACTCGGGCATACCT-3′.
 iNOS (AS): 5′-GGCGAAGAACAATCCACAAC-3′.
 GPx (S): 5′-CGGTTTCCCGTGCAATCAGT-3′.
 GPx (AS): 5′-ACACCGGGGACCAAATGATG-3′.
 GRd (S): 5′-AGCCCACAGCGGAAGTCAAC-3′.
 GRd (AS): 5′-CAATGTAACCGGCACCCACA-3′.
 
*β*-Actin (S): 5′-GGCATCCTGACCCTGAAGTA-3′.
 
*β*-Actin (AS): 5′-GGGGTGTTGAAGGTCTCAAA-3′.

### 2.13. Immunohistochemistry for Detection of MMP-9

For immunohistochemistry, liver sections (4 *μ*m) were deparaffinized and then boiled to unmask antigen sites; the endogenous activity of peroxidase was quenched with 0.03% H_2_O_2_ in absolute methanol. Liver sections were incubated overnight at 4°C with a 1 : 200 dilution of goat polyclonal MMP-9 antibodies (Santa Cruz CA, USA) in phosphate buffered saline (PBS). After removal of the unbound primary antibodies by rinsing with PBS, slides were incubated with a 1 : 500 dilution of biotinylated anti-goat secondary antibody. Bound antibodies were detected with avidin biotinylated peroxidase complex ABC-kit Vectastain and the chromogen 3,3′ diaminobenzidine tetrachloride (DAB) is used as substrate. After appropriate washing in PBS, slides were counterstained with hematoxylin. All sections were incubated under the same conditions with the same concentration of antibodies and at the same time; so the immunostaining was comparable among the different experimental groups.

### 2.14. Statistical Analysis

Differences between obtained values (mean ± SEM) were carried out by one-way analysis of variance (ANOVA) followed by the Duncan test. A *P* value of 0.05 or less was taken as a criterion for a statistically significant difference.

## 3. Results

### 3.1. Phytochemical Screening and GC-MS Findings

Table 1 shows the flavonoids and total polyphenolic contents of* Physalis peruviana* L. fruit juice (physalis juice). Flavonoids content in physalis juice was 89.4 *μ*g/mg quercetin equivalents of flavonoids/mL juice. The total polyphenolic content was 121.3 *μ*g/mg gallic acid equivalent of polyphenols/mL juice. According to the present results, no significant changes were observed between the initial and final flavonoids and phenolics contents indicating the stability of physalis juice.

**Figure 1:**
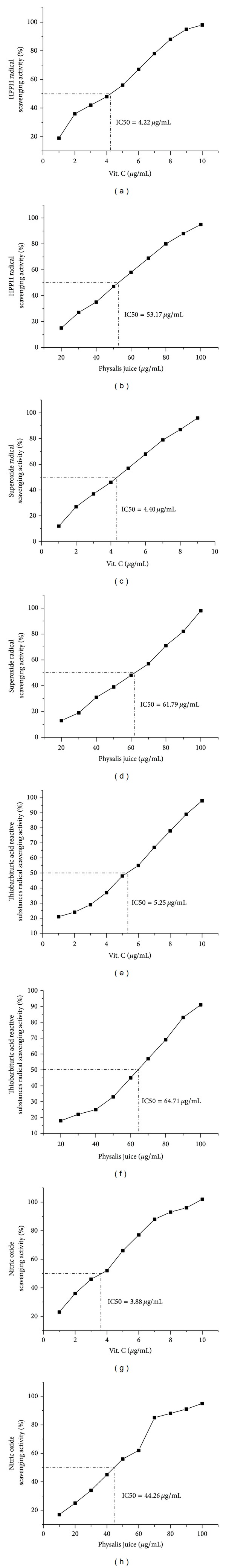
DPPH, superoxide, thiobarbituric acid reactive substances, and nitric oxide radicals scavenging activities of physalis juice and Vit. C. IC 50 values denote the concentration of sample which is required to scavenge 50% of the respective free radicals. Data are represented as mean ± SEM of two independent experiments each performed in triplicate as published in Abdel Moneim and El-Dieb [27].

Figure 1 shows the reduction potential of physalis juice. The order of the reduction potential was physalis juice < Vit. C. Analysis of the free radical scavenging activities of the juice revealed a concentration-dependent antiradical activity resulting from reduction of DPPH^•^, O^•^, TBARS and nitric oxide radicals to nonradical form [27]. The scavenging activity of Vit. C, a known antioxidant, is used as positive control.

**Figure 2:**
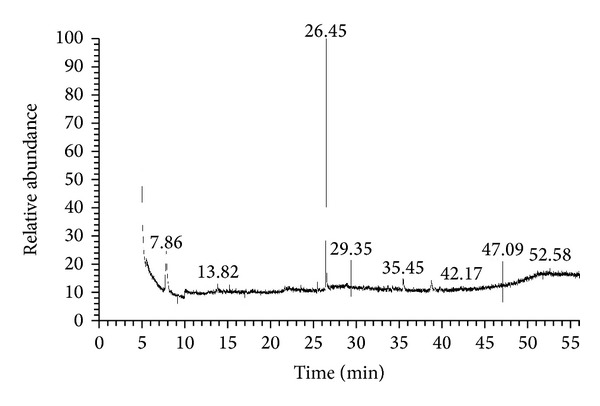
GC-MS chromatogram of physalis juice.

GC-MS chromatogram of the physalis (Figure 2) showed 29 peaks indicating the presence of 29 phytochemical constituents. On comparison of the mass spectra of the constituents with Wiley9 combined with NIST 11 libraries, the 29 phytoconstituents were characterized and identified (Supplementary data, Table S1, in the Supplementary Material available online at http://dx.doi.org/10.1155/2014/381413). The major phytochemical constituent's mass spectra are kaempferol 3-O-rutinoside (1.40%), Quercetin 3,4′,7-trimethyl ether (3.11%), Folic Acid (0.95%), 1,25-Dihydroxyvitamin D2 (1.27%), Lucenin-2 (1.50%), Betulin (0.62%), (5á)Pregnane-3,20á-diol (0.97%).

### 3.2. Histopathologic Findings

**Figure 3:**
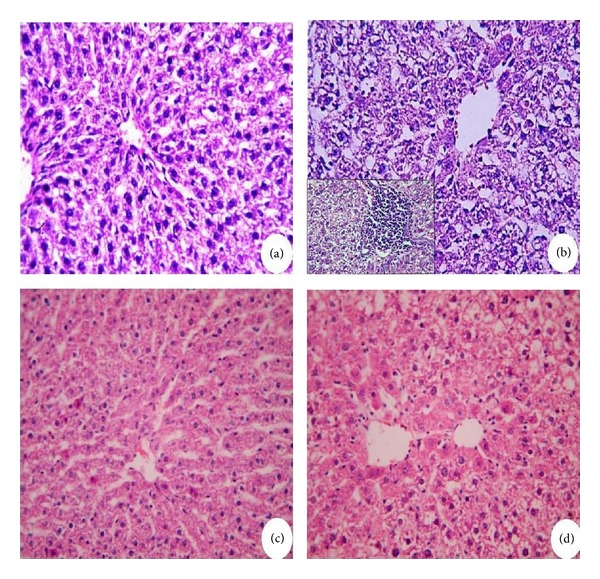
Photomicrographs for liver sections stained with hematoxylin and eosin (H&E). (a) Control rat showing the normal hepatocytes architecture. (b) Rats liver treated with CCl_4_ showing severe hepatic lesions, degenerated and ballooned/necrotic hepatocytes with lymphocyte cells infiltration (corner). (c) Liver treated with physalis similar to control group. (d) Rat liver treated with physalis + CCl_4_ showing maintained hepatic architecture, with minimal damage. Original magnification 400x.

**Figure 4:**
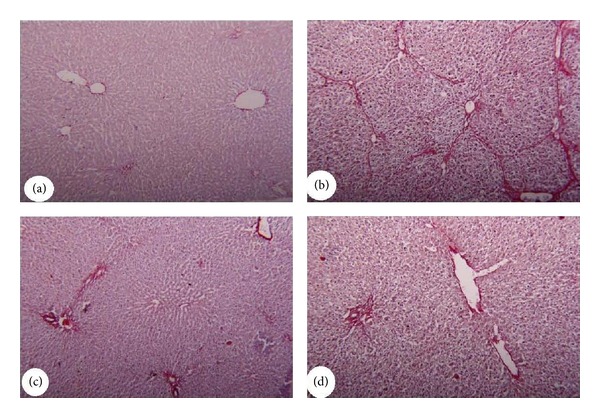
Photomicrographs of liver tissue stained with Sirius Red showing. (a) Control group showing absence of collagen fibers. (b) CCl_4_ group had developed extensive fibrosis in the periportal area and more fibrillar collagen deposition. (c) Physalis group showing absence of collagen fibers. (d) Physalis + CCl_4_ group showing sporadic, small fibrotic lesions in the periportal zone and reduction in collagen fibers deposition. Original magnification 200x.

Control animals showed no abnormality (Figures 3(a) and 4(a)), whereas liver sections of CCl_4_-intoxicated rats showed excessive liver damage with necrosis and inflammation of hepatocytes (Figure 3(b)) and collagen accumulation (Figure 4(b)). Physalis-treated animals showed no abnormalities as well (Figures 3(c) and 4(c)), whereas combined treated groups marked decrease in the liver injury and collagen accumulation as compared with CCl_4_-treated animals (Figures 3(d) and 4(d)).

### 3.3. Biochemical Findings

CCl_4_ injections to the experimental animals caused hepatotoxicity which is indicated by the elevation of the activities of the sera AST, A LT, *γ*-GT, ALP, and TB level, while supplementation of physalis juice exhibited a significant decrease in the levels of these marker enzymes and restored it to the control values as shown in Table 2.

**Figure 5:**
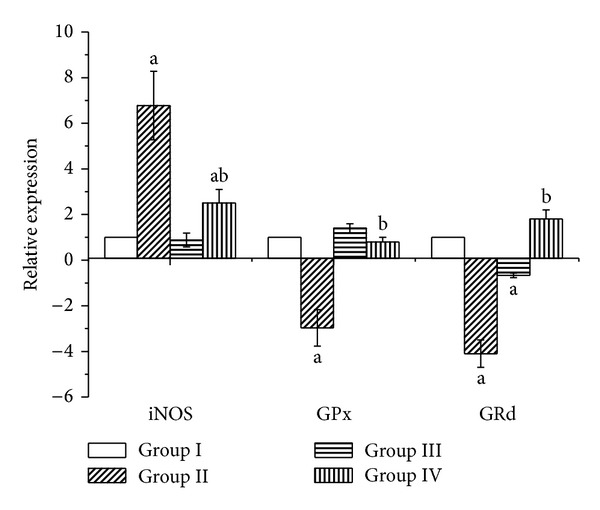
Relative quantification using RT-qPCR of mRNA expression of iNOS, GPx, and GRd genes in liver of rats treated with CCl_4_ and physalis juice. Values are means ± SEM (*n* = 7). ^a^ *P* < 0.05, significant change with respect to Group I; ^b^ *P* < 0.05, significant change with respect to Group II.

MDA, an end product of lipid peroxidation, is widely used as a marker of lipid peroxidation. CCl_4_ injection resulted in a significant increase in MDA in the intoxicated group while treatment withphysalis has significantly reduced the liver injury indicating the hepatoprotective effect of physalis juice (Table 3). The inflammatory marker NO was elevated in the CCl_4_-treated group compared to other groups; this result was further confirmed by the results of mRNA transcripts which have shown increase in the expression of iNOS, while in the treatment group (Group IV) they showed marked reduction in their expression (Figure 5)

Compromised hepatic function is always associated with a state of oxidative stress in liver tissues and serum. Thus, the activities of the redox system were measured in liver tissues. Antioxidant enzymes such as SOD, CAT, GPx, GRd, and GST, as well as glutathione as nonenzymatic antioxidant substance, were estimated in the present study. There was a significant decrease in GSH content in the serum and liver homogenates of CCl_4_-treated groups as compared to the control at *P* < 0.05. The supplementation of physalis with CCl_4_ caused a significant increase in GSH when compared with CCl_4_ group and returned its content to the control level (Table 3). GPx, GRd, and GST activities were also significantly decreased in the liver tissues of rats injected with CCl_4_ (Table 3). The decreases in GPx and GRd were confirmed by results of real time PCR which showed decreases in mRNA expressions by 2.9-fold for GPx and 4-fold for GRd but supplementation with physalis juice was able to significantly ameliorate these enzymes after 12 weeks of treatment concurrently with CCl_4_ (Figure 5).

**Figure 6:**
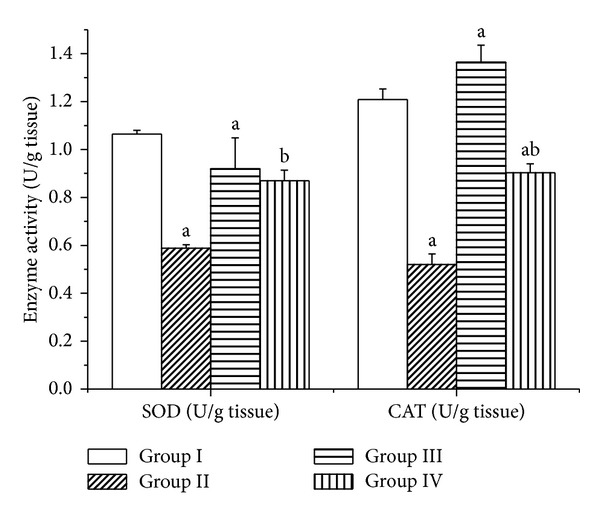
Effects of physalis juice on superoxide dismutase and catalase inhibition in different brain regions of rats treated with CCl_4_. Values are means ± SEM (*n* = 7). ^a^ *P* < 0.05, significant change with respect to Group I; ^b^ *P* < 0.05, significant change with respect to Group II.

Carbon tetrachloride decreased the activities of SOD and CAT, compared to the control group (Figure 6). Rats supplemented withphysalis juice together with CCl_4_ for 12 weeks experienced a significant increase in SOD and CAT activities compared to the CCl_4_-treated group. These improvements indicated thatphysalis juice has antioxidative and beneficial effects for liver recovery from CCl_4_ injury.

### 3.4. Immunohistochemistry Results

**Figure 7:**
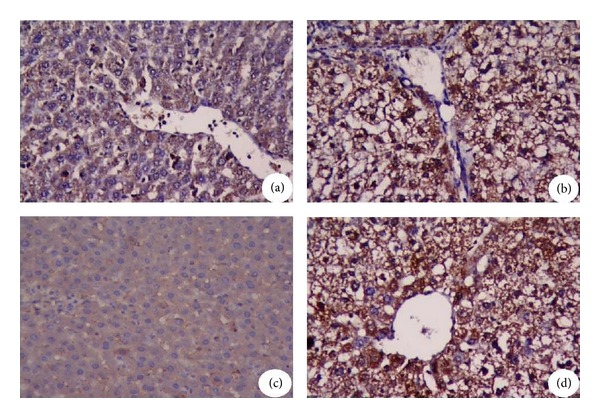
The expression and specific tissue distribution of MMP-9. (a) The liver sections from control rats showed low MMP-9 immunopositive reaction in the hepatic cells. (b) In the livers of rats receiving CCl_4_ for 12 weeks, high MMP-9 expression. (c) Rats receiving physalis juice for 12 weeks show weak immunopositive reaction for MMP-9 expression in hepatic cells. (d) The livers of rats receiving physalis juice concurrently with CCl_4_ showed staining pattern similar or more than to the control animals. Original magnification 400x.

Immunohistochemistry revealed a high expression of MMP-9 in the CCl_4_-treated rats which were reduced in physalis + CCl_4_ group, indicating the antifibrotic effect of physalisjuice (Figure 7(d)).

## 4. Discussion

The oxidative stress induced by CCl_4_, an established model for the evaluation of hepatotoxicity [28–30], was manifested in the hepatic injury observed in the animals treated with CCl_4_. This is due to the CYP P450-mediated metabolism of CCl_4_ to reactive metabolites: CCl_3_^•^ and CCl_3_OO^•^. These radicals bind irreversibly to cellular molecules such as nucleic acid, protein, and lipids, especially the polyunsaturated fatty acids to initiate a process of autocatalytic lipid peroxidation by attacking the methylene bridges of unsaturated fatty acid side chains. This invariably affects the mitochondrial permeability, endoplasmic sequestration, and homeostasis and ultimately in cell damage [31]. Our data show that treatment with CCl_4_ at a dose of 2 mL/kg body weight one time per week for 12 weeks led to the development of hepatic injury and fibrosis in rats. The results obtained in this work are similar to the findings of Ganie et al. [32] and Breikaa et al. [33] who have shown the hepatotoxic effect of CCl_4._

We further tried to evaluate physalis juice hepatoprotection and to show whether it attenuated oxidative stress and inhibited fibrosis in rats treated with CCl_4_. We found that, after supplementation of physalis juice, liver tissue showed a more or less normal lobular pattern with mild degree of necrosis and lymphocytic infiltration almost comparable to normal control.

The general chemical composition of* Physalis peruviana* L. fruit juice has many advantages for its use in medicine [34]. Plant extracts of physalis show antioxidant activity [35] as well as antihepatotoxic [36, 37], antiproliferative effects on hepatoma cells [38] and anti-inflammatory activity [39]. The hepatoprotective effect may be due to presence of quercetin which is one of the main phenolic components present inphysalis and a well-known hepatoprotective agent [30, 40, 41].

A first indication of hepatic damage induced by CCl_4_ was obtained by the evaluation of ALT, AST, ALP, and *γ*-GT. These enzymes have been identified in cytotoxic and cholestatic hepatic injuries. The reversal of these alterations by physalis juice is a clear indication of the improvement of the functional status of hepatocytes with preservation of cellular architecture [42] indicating the hepatoprotective activity of physalis.

MDA was one of the main lipid peroxidation products, its elevated levels could reflect the degrees of lipid peroxidation injury in hepatocytes [43], and administration ofphysalis juice markedly decreases MDA levels indicating its antiperoxidative effect.

NO plays crucial roles in inflammation and liver injury [44]. It is produced in large quantities by Kupffer cells, endothelial cells, and the hepatocytes themselves in response to tissue damage and inflammation induced by various xenobiotics including CCl_4_. In addition, its role in oxidative stress cannot be neglected, since high levels of NO have been associated with oxidative injury via lipid peroxide. Our results showed elevated levels of NO in the CCl_4_ intoxicated rats which is consistent with Breikaa et al. [33]. Our results were supported by the increase in iNOS mRNA gene expression. iNOS is responsible for initiation and propagation of the inflammatory cascade [45];this elevation was significantly decreased after administration of physalis. This may be a potential mechanism by which physalis juice can act as anti-inflammatory and thus protect the liver [39, 46].

Oxidative stress produced by free radicals is the main and primary step in CCl_4_ toxicity contributing to both onset and progression of fibrosis [31]. This was evidenced by enhanced lipid peroxidation, associated with decreased levels of GSH and the antioxidant enzymes system, namely, SOD, CAT, GPx, GRd, and GST.

Many studies reported that these enzymes constitute a mutually supportive team of defense against ROS [47, 48]. An interesting finding in our study is that treatment withphysalis juice attenuated the intoxications of CCl_4_ and significantly improved the activity of these enzymes; this indicates the antioxidant and antihepatotoxic effects of physalis which is similar to results obtained by Arun and Asha [36].

MMP-9 is considered a hallmark of fibrosis whose expression increases by tumor necrosis factor alpha and transforming growth factor beta during the onset of liver fibrogenesis [49]. Immunohistochemical detection in the present study revealed high expression of MMP-9 in the intoxicated rats compared to control group. It has been shown that general inhibitors of MMPs attenuate hepatic fibrosis or are useful to inhibit acute and chronic inflammatory or vascular diseases [50].

Another interesting finding of our study is that treatment withphysalis juice has downregulated the expression of MMP-9 and thus inhibiting fibrosis. The antioxidant and antifibrotic effect of physalis may be due to presence of quercetin [41]. Our GC-MS results have shown that it contains high amount of it. Quercetin belongs to an extensive class of polyphenolic flavonoid compounds almost ubiquitous in plants and plant food sources. Quercetin is considered to be a strong antioxidant due to its ability to scavenge free radicals and bind transition metal ions. These properties of quercetin allow it to inhibit lipid peroxidation [51] and have anti-inflammatory properties [52]. In addition to quercetin, physalis juice also contains kaempferol 3-O-rutinoside. Kaempferol is known to be potential antioxidant due to its ability to scavenge free radicals and active oxygen species such as singlet oxygen, superoxide anion radical, and hydroxyl radicals [53]. The presence of these compounds could explain the antioxidant activity found in the crude extract.

## 5. Conclusion

The findings of the present study indicate the hepatoprotective effect of physalis on CCl_4_ intoxicated rats. This was accompanied with improvements in liver function, decreasing collagen areas and hepatic fibrosis. The underlying mechanisms for this hepatoprotection may be due scavenging free radicals, as well as decreasing the expression of the well-known fibrotic marker MMP-9. Thus blocking the oxidative stress pathway may be of therapeutic value in treatment of liver injury and fibrosis.

## Supplementary Material

GC-MS chromatogram of the physalis showed 29 peaks indicating the presence of 29 phytochemical constituents. On comparison of the mass spectra of the constituents with Wiley9 combined with NIST 11 libraries, the 29 phytoconstituents were characterized and identified as it shown in Table S1. The identified phytochemical constituent's mass spectra are Oleic acid, eicosyl ester (5.04%), Colchifoleine (1.48 %), 3*β*,11*β*,21-Trihydroxy-20-oxo-5*α*-pregnan-18-oic acid 18,11-lactone (0.63%), 1-Hydroxy-2-(2,3,4,6-tetra-O-acetyl-beta-D-glucopyranosyl)-9H-xanthene-3,6,7-triyl triacetate (0.81%), kaempferol 3-O-rutinoside (1.40%), 25*β*-Cholan-24-oic acid, 3,12-dioxo- (2.58%), alpha-D-Glucopyranoside, methyl 2-(acetylamino)-2-deoxy-3-O-(trimethylsilyl)-, cyclic methylboronate (1.62%), ethyl iso-allocholate (1.99%), Quercetin 3,4',7-trimethyl ether (3.11%), Folic Acid (0.95%), 1,25-Dihydroxyvitamin D2 (1.27%), Docosane (0.93), 3-Hydroxy-4,4-dimethyl-7-oxoandrost-5-en-17-yl acetate (0.71%), (5*β*)Pregnane-3,20*β*-diol, 14*α*,18*α*-[4-methyl-3-oxo-(1-oxa-4-azabutane-1,4-diyl)], diacetate (1.45%), Pregna-4,6-diene-21-carboxylic acid, 17-hydroxy-3-oxo-, *γ*-lactone (0.75%), Hexadecatrienoic acid, methyl ester (0.64%), beta-k-strophanthin (2.48%), 2,2,4,9,11,11-Hexamethyl dodecane (0.66%), Cholest-5-en-3-one (0.63%), 9,12,15-Octadecatrienoic acid (2-phenyl-1,3-dioxolan-4-yl)methyl ester (0.89%), 9-cis-Hexadecenoic acid (0.93%), 3,7,11-Trihydroxypregnan-20-one (0.99%), Ceanothine C (1.26%), Methyl-9,9,10,10-D4-octadecanoate (4.01%), Lucenin-2 (1.50%), Betulin (0.62%), (5á)Pregnane-3,20á-diol (0.97%), Anodendroside G, monoacetate (0.64%) and 7,8-Epoxylanostan-11-ol, 3-acetoxy- (0.61%).

## Figures and Tables

**Table 1:** Total flavonoids and polyphenolic contents of physalis juice in different conditions.

Conditions	Total phenolics^a^	Total flavonoid^b^
Physalis juice, fresh	121.3 ± 4.65	89.4 ± 4.82
Physalis juice, store*	113.5 ± 5.31	81.7 ± 3.74

^a^Flavonoids are expressed as *μ*g/mg quercetin equivalents of flavonoids/mL juice. ^b^Total polyphenols are expressed as *μ*g/mg gallic acid equivalent of polyphenols/mL juice. Data are represented as mean ± SEM of two independent experiments each performed in duplicate. *Stored at room temperature for 3 days.

**Table 2:** Serum levels of liver enzymes in different studied groups.

Parameters	Group I	Group II	Group III	Group IV
GPT (U/mL)	70.12 ± 1.88	153.91 ± 1.56^a^	68.47 ± 1.23	79.10 ± 5.12^b^
GOT (U/mL)	56.08 ± 1.30	123.75 ± 7.21^a^	56.33 ± 1.21	57.08 ± 1.45^b^
*γ* -GT (U/L)	38.77 ± 2.66	52.80 ± 1.60^a^	34.87 ± 1.21	35.39 ± 0.56^b^
ALP (IU/L)	147.73 ± 4.07	244.32 ± 7.52^a^	116.48 ± 5.31^a^	165.28 ± 7.10^b^
TB (mg/dL)	2.64 ± 0.08	4.19 ± 0.08^a^	2.81 ± 0.05	3.20 ± 0.09^ab^

Values are means ± SEM (*n* = 7).

^a^ *P* < 0.05, significant change with respect to Group I; ^b^ *P* < 0.05, significant change with respect to Group II.

**Table 3:** Sera MDA, NO, and GSH and hepatic MDA, NO, GSH, GRd, GST, and GPx in different studied groups.

Parameters	Group I	Group II	Group III	Group IV
Serum MDA (nmol/mL)	32.37 ± 0.61	50.21 ± 1.46^a^	23.18 ± 1.51^a^	37.52 ± 1.38^b^
Serum NO (*μ*mol/L)	47.33 ± 1.64	101.13 ± 4.79^a^	52.30 ± 2.99	80.10 ± 2.10^ab^
Serum GSH (mmol/mL)	53.02 ± 2.86	39.13 ± 1.31^a^	72.00 ± 4.91^a^	63.46 ± 3.65^ab^
Hepatic MDA (nmol/g tissue)	427.19 ± 4.79	591.45 ± 12.47^a^	360.75 ± 7.85^a^	406.82 ± 2.80^b^
Hepatic NO (*μ*mol/g tissue)	128.54 ± 2.39	226.50 ± 2.93^a^	139.33 ± 6.82	184.59 ± 8.67^ab^
Liver GSH (mmol/g tissue)	36.69 ± 2.01	25.09 ± 1.47^a^	55.51 ± 2.73^a^	44.16 ± 5.66^b^
Hepatic GRd (*μ*mol/g tissue)	102.48 ± 8.97	41.5297 ± 3.15^a^	105.16 ± 10.95	86.4085 ± 5.75^b^
Hepatic GST (*μ*mol/h/g tissue)	0.27 ± 0.03	0.12 ± 0.01^a^	0.31 ± 0.02	0.22 ± 0.03^ab^
Hepatic GPx (U/g tissue)	1722.43 ± 69.21	616.02 ± 27.76^a^	2054.37 ± 54.68^a^	1532.68 ± 38.97^b^

Values are means ± SEM (*n* = 7).

^a^ *P* < 0.05, significant change with respect to Group I; ^b^ *P* < 0.05, significant change with respect to Group II.
