# Supplementary material for: The Potential Protective Effect of Physalis peruviana L. against Carbon Tetrachloride-Induced Hepatotoxicity in Rats Is Mediated by Suppression of Oxidative Stress and Downregulation of MMP-9 Expression
Source: Oxid Med Cell Longev. 2014 Apr 27;2014:381413. doi: 10.1155/2014/381413 (PMC4020166; doi:10.1155/2014/381413)
Supplement: Supplementary file 1 — GC-MS chromatogram of the physalis showed 29 peaks indicating the presence of 29 phytochemical constituents. On comparison of the mass spectra of the constituents with Wiley9 combined with NIST 11 libraries, the 29 phytoconstituents were characterized and identified as it shown in Table S1. The identified phytochemical constituent's mass spectra are Oleic acid, eicosyl ester (5.04%), Colchifoleine (1.48 %), 3β,11β,21-Trihydroxy-20-oxo-5α-pregnan-18-oic acid 18,11-lactone (0.63%), 1-Hydroxy-2-(2,3,4,6-tetra-O-acetyl-beta-D-glucopyranosyl)-9H-xanthene-3,6,7-triyl triacetate (0.81%), kaempferol 3-O-rutinoside (1.40%), 25β-Cholan-24-oic acid, 3,12-dioxo- (2.58%), alpha-D-Glucopyranoside, methyl 2-(acetylamino)-2-deoxy-3-O-(trimethylsilyl)-, cyclic methylboronate (1.62%), ethyl iso-allocholate (1.99%), Quercetin 3,4',7-trimethyl ether (3.11%), Folic Acid (0.95%), 1,25-Dihydroxyvitamin D2 (1.27%), Docosane (0.93), 3-Hydroxy-4,4-dimethyl-7-oxoandrost-5-en-17-yl acetate (0.71%), (5β)Pregnane-3,20β-diol, 14α,18α-[4-methyl-3-oxo-(1-oxa-4-azabutane-1,4-diyl)], diacetate (1.45%), Pregna-4,6-diene-21-carboxylic acid, 17-hydroxy-3-oxo-, γ-lactone (0.75%), Hexadecatrienoic acid, methyl ester (0.64%), beta-k-strophanthin (2.48%), 2,2,4,9,11,11-Hexamethyl dodecane (0.66%), Cholest-5-en-3-one (0.63%), 9,12,15-Octadecatrienoic acid (2-phenyl-1,3-dioxolan-4-yl)methyl ester (0.89%), 9-cis-Hexadecenoic acid (0.93%), 3,7,11-Trihydroxypregnan-20-one (0.99%), Ceanothine C (1.26%), Methyl-9,9,10,10-D4-octadecanoate (4.01%), Lucenin-2 (1.50%), Betulin (0.62%), (5á)Pregnane-3,20á-diol (0.97%), Anodendroside G, monoacetate (0.64%) and 7,8-Epoxylanostan-11-ol, 3-acetoxy- (0.61%). [file 381413.f1.pdf]

Table S1: Phytochemicals compounds identified in *Physalis peruviana* juice by GC-MS.

Peak	Retention time	Name of the compound	Molecular formula	Molecular weight	Area %
**1**	5.50 min	Oleic acid, eicosyl ester	$C_{38}H_{76}O_2$	562	5.04
**2**	6.24 min	Colchifoleine	$C_{21}H_{23}NO_7$	401	1.48
**3**	6.40 min	3β,11β,21-Trihydroxy-20-oxo-5α-pregnan-18-oic acid 18,11-lactone	$C_{21}H_{30}O_5$	362	0.63

**4**	9.12 min	1-Hydroxy-2-(2,3,4,6-tetra-O-acetyl-beta-D-glucopyranosyl)-9H-xanthene-3,6,7-triyl triacetate	$C_{33}H_{34}O_{18}$	718	0.81
**5**	9.18 min	kaempferol 3-O-rutinoside	$C_{27}H_{30}O_{15}$	594	1.40
**6**	10.03 min	3,12-Diketocholanic acid	$C_{24}H_{36}O_4$	388	2.58
**7**	10.11 min	alpha-D-Glucopyranoside, methyl 2-(acetylamino)-2-deoxy-3-O-(trimethylsilyl)-, cyclic methylboronate	$C_{13}H_{26}BNO_6Si$	331	1.62
**8**	13.67 min	ethyl iso-allocholate	$C_{26}H_{44}O_5$	436	1.99
**9**	13.83 min	Quercetin 3,4',7-trimethyl ether	$C_{18}H_{16}O_7$	344	3.11
**10**	14.00 min	Folic Acid	$C_{19}H_{19}N_7O_6$	441	0.95
**11**	14.84 min	1,25-Dihydroxyvitamin D2	$C_{28}H_{44}O_3$	428	1.27
**12**	15.20 min	Docosane	$C_{22}H_{46}$	310	0.93
**13**	17.00 min	3-Hydroxy-4,4-dimethyl-7-oxoandrost-5-en-17-yl acetate	$C_{23}H_{34}O_4$	374	0.71
**14**	17.07 min	(5β)Pregnane-3,20β-diol, 14α,18α-[4-methyl-3-oxo-(1-oxa-4-azabutane-1,4-diyl)], diacetate	$C_{28}H_{43}NO_6$	489	1.45
**15**	21.66 min	Pregna-4,6-diene-21-carboxylic acid, 17-hydroxy-3-oxo-, γ-lactone	$C_{22}H_{28}O_3$	340	0.75
**16**	24.96 min	Hexadecatrienoic acid, methyl ester	$C_{17}H_{28}O_2$	264	0.64

**17**	25.43 min	beta-k-strophanthin	$C_{36}H_{54}O_{14}$	710	2.48
**18**	27.07 min	2,2,4,9,11,11-Hexamethyl dodecane	$C_{18}H_{38}$	254	0.66
**19**	28.24 min	Cholest-5-en-3-one	$C_{27}H_{44}O$	384	0.63
**20**	29.35 min.	9,12,15-Octadecatrienoic acid (2-phenyl-1,3-dioxolan-4-yl)methyl ester	$C_{28}H_{40}O_4$	440	0.89
**21**	31.34 min	9-cis-Hexadecenoic acid	$C_{34}H_{64}O_2$	504	0.93
**22**	33.63 min	3,7,11-Trihydroxypregnan-20-one	$C_{21}H_{34}O_4$	350	0.99
**23**	34.16 min	Ceanothine C	$C_{26}H_{38}N_4O_4$	470	1.26
**24**	35.42 min	Methyl-9,9,10,10-D4-octadecanoate	$C_{19}H_{34}D_4O_2$	298	4.01
**25**	39.21 min	Lucenin-2	$C_{27}H_{30}O_{16}$	610	1.50
**26**	39.73 min	Betulin	$C_{30}H_{50}O_2$	442	0.62
**27**	47.70 min	(5á)Pregnane-3,20á-diol	$C_{28}H_{43}NO_6$	489	0.97
**28**	48.35 min	Anodendroside G, monoacetate	$C_{32}H_{42}O_{11}$	602	0.64
**29**	54.18 min	7,8-Epoxylanostan-11-ol, 3-acetoxy-	$C_{32}H_{54}O_4$	502	0.61
